# Pivoting Method of Inserting Artificial Teeth

**Published:** 1840-07

**Authors:** 


### PIVOTING METHOD OF INSERTING ARTIFICIAL TEETH.

Our object in adverting to this subject, is merely to notice a plan proposed by Mr. L. S. Stringfellow, an ingenious and skilful practitioner, for giving egress to matter, whenever formed, within the root of a tooth on which an artificial crown has been placed, and by which the supervention of alveolar abscess may, in almost every instance be prevented. For this purpose, instead of cutting a groove on the side of the pivot, as recommended some years ago by Dr. L. S. Parmly, of New Orleans, he cuts it on the side of the tooth; and has invented an instrument by which it can be done with very great facility. This, together with several other ingeniously constructed instruments invented by Mr. S. for the excavation of carious portions of teeth, preparatory to filling, we may take occasion to describe in a future number of our Journal.—BALT. ED.

